# Feasibility of artificial intelligence assisted quantitative muscle ultrasound in carpal tunnel syndrome

**DOI:** 10.1186/s12891-023-06623-3

**Published:** 2023-06-27

**Authors:** Sun Woong Kim, Sunwoo Kim, Dongik Shin, Jae Hyeong Choi, Jung Sub Sim, Seungjun Baek, Joon Shik Yoon

**Affiliations:** 1 Department of Physical and Rehabilitation Medicine, Korea University Guro Hospital, 148 Gurodong-ro, Guro-gu, Seoul, 08308 Republic of Korea; 2 Department of Computer Science and Engineering, Korea University, 145, Anam-ro, Seoul, 02841 Republic of Korea

**Keywords:** Muscle ultrasound, Quantitative ultrasound, Carpal tunnel syndrome, Machine learning, Artificial intelligence

## Abstract

**Background:**

In case of focal neuropathy, the muscle fibers innervated by the corresponding nerves are replaced with fat or fibrous tissue due to denervation, which results in increased echo intensity (EI) on ultrasonography. EI analysis can be conducted quantitatively using gray scale analysis. Mean value of pixel brightness of muscle image defined as EI. However, the accuracy achieved by using this parameter alone to differentiate between normal and abnormal muscles is limited. Recently, attempts have been made to increase the accuracy using artificial intelligence (AI) in the analysis of muscle ultrasound images. CTS is the most common disease among focal neuropathy. In this study, we aimed to verify the utility of AI assisted quantitative analysis of muscle ultrasound in CTS.

**Methods:**

This is retrospective study that used data from adult who underwent ultrasonographic examination of hand muscles. The patient with CTS confirmed by electromyography and subjects without CTS were included. Ultrasound images of the unaffected hands of patients or subjects without CTS were used as controls. Ultrasonography was performed by one physician in same sonographic settings. Both conventional quantitative grayscale analysis and machine learning (ML) analysis were performed for comparison.

**Results:**

A total of 47 hands with CTS and 27 control hands were analyzed. On conventional quantitative analysis, mean EI ratio (i.e. mean thenar EI/mean hypothenar EI ratio) were significantly higher in the patient group than in the control group, and the AUC was 0.76 in ROC analysis. In the analysis using machine learning, the AUC was the highest for the linear support vector classifier (AUC = 0.86). When recursive feature elimination was applied to the classifier, the AUC value improved to 0.89.

**Conclusion:**

This study showed a significant increase in diagnostic accuracy when AI was used for quantitative analysis of muscle ultrasonography. If an analysis protocol using machine learning can be established and mounted on an ultrasound machine, a noninvasive and non-time-consuming muscle ultrasound examination can be conducted as an ancillary tool for diagnosis.

**Supplementary Information:**

The online version contains supplementary material available at 10.1186/s12891-023-06623-3.

## Background

In the case of focal neuropathy, the muscle fibers innervated by the corresponding nerves are replaced with fat or fibrosis due to denervation, which results in increased echo intensity (EI) on ultrasonography [1]. EI analysis can be performed visually, qualitatively, and quantitatively. In the case of quantitative analysis, grayscale analysis is performed. In this analysis, parameters such as mean and standard deviation (SD) are mainly used; however, the attainable accuracy achieved by using this parameter alone to differentiate between normal and abnormal muscles is limited [2, 3]. Recently, attempts have been made to increase the accuracy of diagnosis using artificial intelligence (AI) in the analysis of muscle ultrasound images. However, it was studied in generalized neuropathy [4].

Carpal tunnel syndrome (CTS) is a disease that not only causes numbness in the hand as the median nerve is compressed at the wrist, but also causes weakness in the hand muscles in severe cases. It is the most common focal compressive neuropathy, with a prevalence of approximately 3% in the general population [5]. Therefore, its diagnosis and appropriate treatment are very important for clinicians. However, no attempts have been made to increase the accuracy of distinguishing the hand with CTS and without using AI assisted quantitative muscle ultrasound analysis. The purpose of this study was to verify the utility of AI-assisted quantitative muscle ultrasound analysis in CTS.

## Materials and methods

### Subjects

This was a retrospective study that used data from adult who underwent ultrasonographic examination of the hand muscles at the Department of Physical Medicine and Rehabilitation, University Hospital. The patient with CTS confirmed by electromyography and subjects without CTS were included. Electromyographic diagnosis and grading complied with the American Association of Neuromuscular and Electrodiagnostic Medicine (AANEM) guidelines[6, 7]. Ultrasound images of the unaffected hands of patients and the subjects without CTS were used as controls. Patients with myelopathy, myopathy, neuromuscular junction disease, motor cell disease, cervical radiculopathy, ulnar neuropathy, and other systemic peripheral neuropathies were not included. This study was approved by the Institutional Review Board of the relevant institution (IRB No. 2021GR0506).

### Muscle ultrasound

Ultrasonography was performed by one experienced rehabilitation physician using the HD15 system (Philips Ultrasound, Bothell, WA, USA) with a linear array transducer. Muscle ultrasonography was performed on the thenar muscles innervated by the median nerve and hypothenar muscles innervated by the ulnar nerve. The transverse muscle image was acquired around midpoint of the first (i.e. thenar muscle) or 5th metatarsal bone (i.e. hypothenar muscle) by placing the transducer perpendicular to the bone (Total 74 hands). Of total 74 hands, in the 53 hands, images were only acquired around midpoint of thenar and hypothenar muscle. In another 17 hands, images were additionally acquired from 9 different sites other than the midpoint of muscles in the thenar and hypothenar, respectively. In the other 4 hands, images were additionally obtained from 10 different sites other than the midpoint of each muscles. These additional images which acquired from sites other than the midpoint were used only in the analysis of machine learning not in conventional quantitative analysis. The summary of our dataset is presented in [Table 1]. The participants were instructed to fully relax their hand muscles in forearm supination, wrist-neutral, and elbow extension positions. Ultrasound gain, dynamic range, depth, and transducer frequency settings were kept constant for all image acquisitions throughout the study (gain = 54, dynamic range = 59, depth = 3 cm, frequency = 65 Hz).

**Table 1:** Dataset of ultrasound images (number of hands) for the machine learning analysis

Label	Severity	Dataset	
		Data for ML Training	Data for ML Test
Normal	Normal	104 (19)	26 (8)
CTS	Mild	31 (4)	8 (8)
	Moderate	62 (16)	15 (7)
	Severe	17 (8)	4 (4)
Total		214 (47)	53 (27)

CTS, carpal tunnel syndrome. Data presented above means number of image pairs (number of hands). A pair of images consists of thenar and hypothenar images, and a total of 267 pairs of images were obtained from 74 hands

### Data analysis

#### Conventional quantitative analysis

**Fig. 1:**
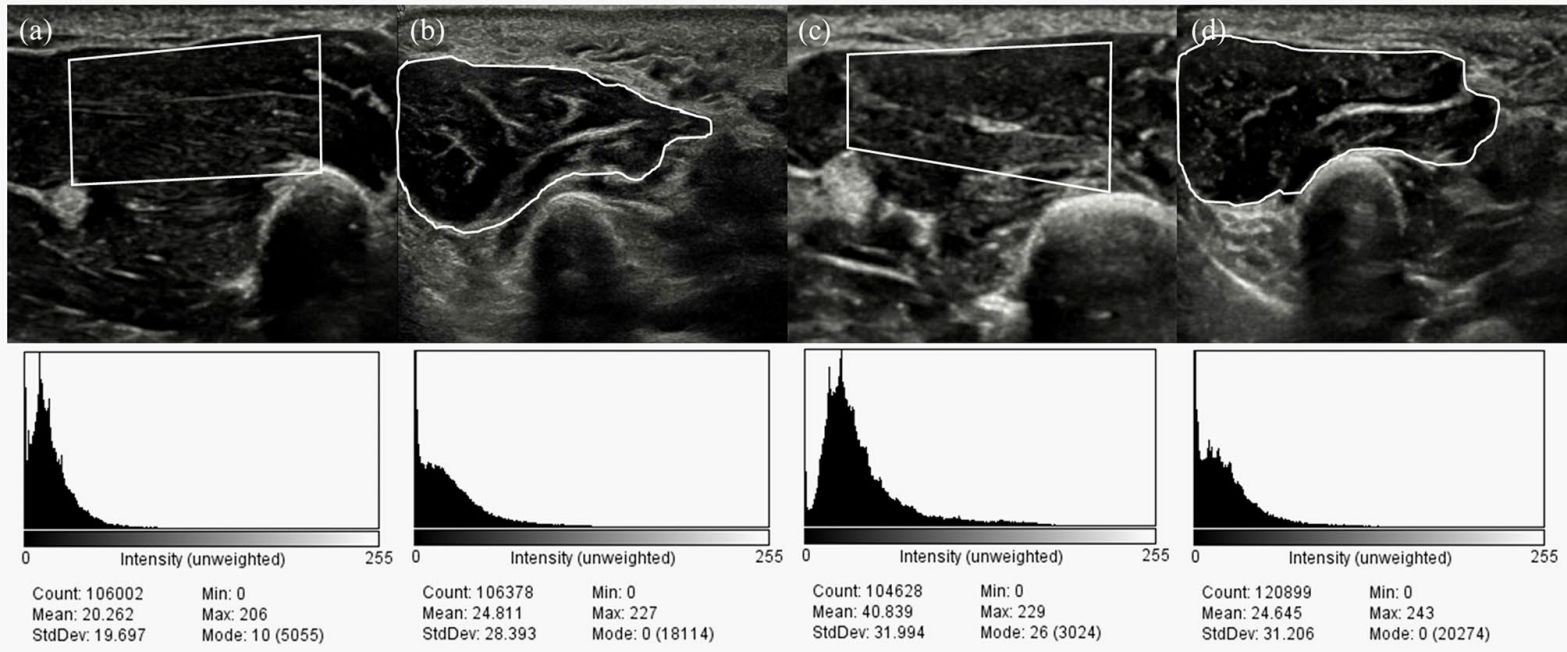
Representative US images of hand muscles and derived histograms. White rectangular or free hand regions of US images is region of interest (ROI). Mean and StdDev is the mean and standard deviation of pixel brightness in ROI. [**a**-**b**] Thenar muscle (**a**) and the hypothenar muscle (**b**) in the control group. [c-d] The thenar muscle (**c**) and hypothenar muscle (**d**) of a patient with moderate-degree CTS

In the ultrasound images which was obtained around midpoint of the first (i.e. thenar muscle) or 5th metatarsal bone (i.e. hypothenar muscle), the region of interest (ROI) was set manually using the ImageJ software (Wayne Rasband, Kensington, MD) by physician. The ROI of the thenar muscle was set by using four boundaries (outer, inner, lower, upper) so that only the muscles innervated by median nerve is included [Fig. 1] [8]. The ROI of the hypothenar muscle was set by tracing the border of the hypoechoic area of muscle [Fig. 1] [8]. Quantitative grayscale analysis of EI was performed automatically in program using the gray-level intensity histogram. The mean and SD of EI were obtained for the thenar and hypothenar muscles, respectively. The mean EI ratio of two muscles was attained by dividing the mean EI value of the thenar muscle by the mean EI value of the hypothenar muscle. This mean EI value was compared between hands with CTS and without.

#### AI analysis

**Fig. 2:**
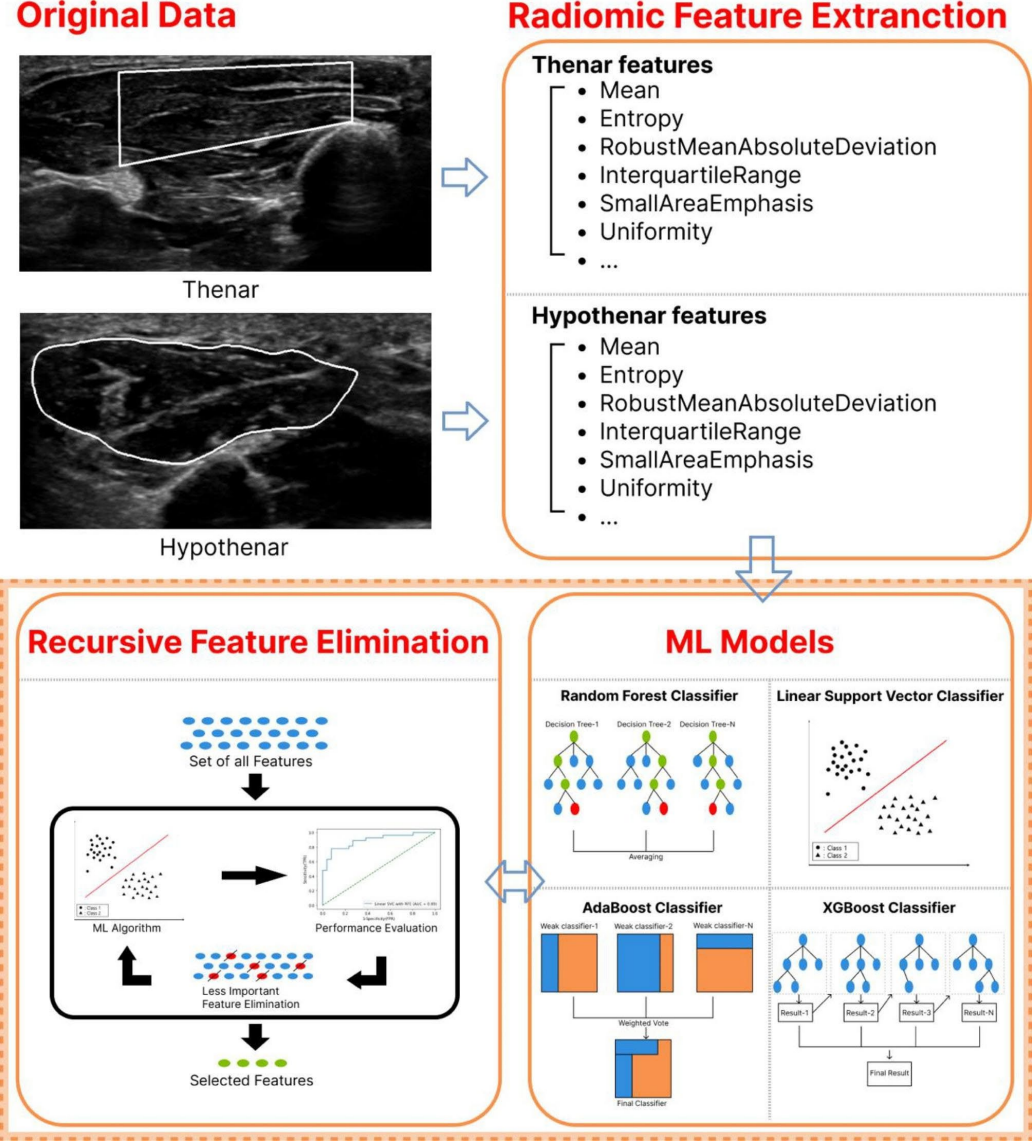
Overview of our approach. We applied machine learning (ML) algorithms to improve prediction accuracy and to identify important features required for classification. We extracted radiomic features from the regions of interests (ROI) of thenar and hypothenar, then predicted performance using four ML classifiers. In addition, important features were selected by applying recursive feature elimination (RFE) to each classifier

We present a binary classification analysis using several features obtained from muscle ultrasound images. Several features were obtained from Pyradiomics, an open-source platform capable of extracting a group of formulated features from medical images [9]. A total of 176 radiomic features, 88 each for the thenar and hypothenar areas were extracted and used as the input data to the machine learning (ML) classifiers for the classification of patients with CTS. We considered the following ML classifiers: (a) random forest, (b) adaptive boosting (AdaBoost), (c) support vector classifier (SVC), and (d) extreme gradient boosting (XGB). The dataset was split into the training and test sets at a ratio of 80:20. The training and test images were obtained from independent patient groups. That is, the subjects of images used for machine learning ‘training’ and the subjects of images used for machine learning ‘test’ do not overlap each other. [Table 1] summarizes the datasets. Also, our experiment was conducted with five-fold cross-validation at each iteration, i.e., 80% of the training data were used for training and the remaining 20% were used for validation. We evaluated the classification performance using the following metrics: area under the ROC curve (AUC), precision, recall, and F1 score. Additionally, we performed recursive feature elimination (RFE) to identify useful features for the diagnosis of CTS form among the 88 features. RFE is a method for removing features of low importance during training until the desired number of features remains [10]. We performed RFE for each of the four classifiers, and the same performance metric was used after the feature was selected by applying RFE. The overall metric was calculated as the average of repeated 100 times experiments for the test dataset. [Figure 2] outlines the overall approach to the AI analysis.

### Statistical analysis in conventional analysis

Data were analyzed using SPSS version 26.0 software. Mann-Whitney U test was used to investigate whether there was a significant difference in the echo-intensity of thenar or hypothenar muscle and ratio of echo-intensity (thenar/hypothenar) between the hands with CTS and without Statistical significance was set at p-value < 0.05. ROC curve analysis was performed to set cutoff values and to calculate sensitivity and specificity.

## Results

This study analyzed 137 images of hands with CTS (N = 47) and 130 images of control hands (N = 27). When categorizing respect to severity, 12 were with mild; 23, moderate; and 12, severe. Baseline characteristics is provided in supplementary Table 1. The group of hands with CTS was older than the group without CTS, and there was no significant difference in gender and hand dominance between the two groups.

### Conventional quantitative analysis

**Fig. 3:**
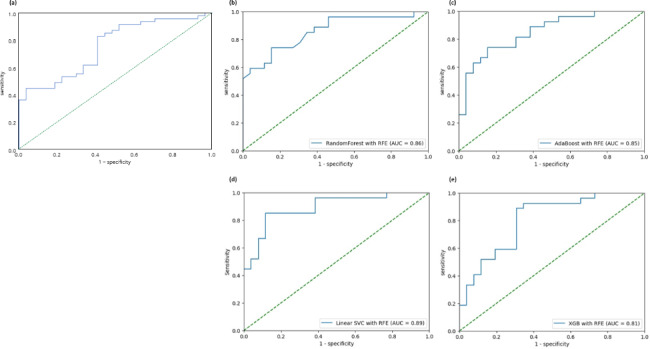
ROC curve and AUC score. (**a**) ROC using mean echo-intensity ratio (thenar/hypothenar) for distinguishing the hands with CTS. (**b** – **e**) ROC for each machine learning (ML) classifier. RFE was not applied to XGB classifier since its performance was better without RFE. (**b**) Random forest. (**c**) Adaptive boosting. (**d**) Linear support vector classifier. (**e**) XGB classifier

When quantitative analysis was performed, the mean thenar EI was 38.37 ± 14.80 in hands with CTS and 32.03 ± 7.03 in the control hands. The mean hypothenar EI was 28.23 ± 13.03 in hands with CTS and 30.16 ± 7.86 in the control hands. In Mann-Whitney U test, EI of thenar and hypothenar muscle had no significant difference between hands with CTS and without (p = 0.08, 0.07, respectively). The mean EI ratio (i.e. mean EI value of the thenar muscle/mean EI value of the hypothenar muscle) was 1.43 ± 0.39 and median value was 1.33(1.16–1.63) in the hands with CTS. In the control hands, the mean EI ratio 1.10 ± 0.24 and the median value was 1.09(0.85–1.31) in the control hands. The mean value of SD ratio (i.e. SD of the thenar muscle/SD of the hypothenar muscle) was 1.02 ± 0.24 and the median value was 0.99(0.87–1.17) in the hands with CTS. In the control hands, the mean SD was 0.82 ± 0.15 and the median value was 0.81(0.72–09.96). In Mann-Whitney U test, the hands with CTS showed higher mean thenar/hypothenar EI or SD ratio (p < 0.001, respectively). In ROC analysis of the EI ratio, the AUC was 0.755. The sensitivity and specificity were 83.0% and 59.3% in the cut off value of 1.422. [(a) of Fig. 3]

### AI-assisted quantitative analysis

[Table 2] describes the results of the classifiers and application of RFE to each classifier. [(b) – (e) of Fig. 3] shows the ROC curve and AUC for each classifier. RFE was applied to all ML classifiers excepted XGB classifier because its performance was better in without. All classifiers showed high AUC values of ≥ 0.80 without RFE, particularly in the case of SVC using a linear kernel close to 0.86. In addition, the linear SVC showed the highest performance in precision and F1 score of 0.77 and 0.82, respectively. Furthermore, the random forest classifier had the highest recall score of 0.95.

**Table 2:** Performance evaluation of each classifier

Classifier	Performance metric			
	AUC	Precision	Recall	F1 score
Random forest	0.83 (0.008)	0.66 (0.008)	*0.95 (0.010)*	0.77 (0.007)
Random forest with RFE	*0.86 (0.006)*	0.66 (0.010)	**0.95 (0.009)**	0.78 (0.008)
AdaBoost	0.80 (0.012)	0.70 (0.016)	0.86 (0.022)	0.77 (0.015)
AdaBoost with RFE	0.85 (0.010)	0.72 (0.015)	0.88 (0.017)	0.79 (0.012)
Linear SVC	*0.86 (0.007)*	**0.77 (0.015)**	0.88 (0.013)	**0.82 (0.011)**
Linear SVC with RFE	**0.89 (0.002)**	*0.75 (0.008)*	0.88 (0.007)	*0.81 (0.005)*
XGB	0.81 (0.008)	0.66 (0.009)	0.89 (0.015)	0.76 (0.009)
XGB with RFE	0.79 (0.008)	0.65 (0.009)	0.86 (0.011)	0.74 (0.008)

AUC, area under the ROC curve; AdaBoost, adaptive boosting; SVC, support vector classifier; XGB, extreme gradient boosting; RFE, recursive feature elimination. Data are presented as performance of test data (standard derivative). The scores in bold are the best performance, and those in italics with underlines are the second best

When removing features of low importance (RFE), the thenar and hypothenar muscles were removed as pairs. As a result, a total of 30 features (15 each from the thenar and hypothenar areas) were selected. Compared with the case of using all features, in the case of the XGB classifier, the AUC score slightly decreased from 0.81 to 0.79. However, the AUC score increased for other classifiers. There were commonly selected features in three or more of the four classifiers, which are as follows: interquartile range (IQR), robust mean absolute deviation (rMAD), and small-area emphasis. These features were deemed important for classification. We will discuss these features in the following section. [Table 3] summarizes the results.

**Table 3:** RFE feature selection

Classifier	AUC	Selected Features
Random Forest	0.86	MedianAbsoluteDeviation, Entropy, **InterquartileRange**, Kurtosis, MeanAbsoluteDeviation, **RobustMeanAbsoluteDeviation**, Uniformity, JointEnergy, JointEntropy, SumEntropy, LargeDependenceHighGrayLevelEmphasis, LowGrayLevelRunEmphasis, RunVariance, LowGrayLevelZoneEmphasis, **SmallAreaEmphasis**
AdaBoost	0.85	**InterquartileRange**, MeanAbsoluteDeviation, Mean, **RobustMeanAbsoluteDeviation**, TotalEnergy, Uniformity, Variance, Autocorrelation, Idmn, Idn, MaximumProbability, HighGrayLevelRunEmphasis, LowGrayLevelZoneEmphasis, SizeZoneNonUniformity, **SmallAreaEmphasis**
SVC	0.89	MedianAbsoluteDeviation, 90Percentile, **RobustMeanAbsoluteDeviation**, Autocorrelation, Imc1, JointAverage, JointEntropy, MaximumProbability, SumAverage, SumEntropy, DependenceVariance, LargeDependenceHighGrayLevelEmphasis, LargeDependenceLowGrayLevelEmphasis, RunEntropy, **SmallAreaEmphasis**
XGB	0.79	MedianAbsoluteDeviation, Energy, **InterquartileRange**, Uniformity, Autocorrelation, Idn, Imc1, LongRunLowGrayLevelEmphasis, LowGrayLevelRunEmphasis, RunLengthNonUniformityNormalized, ShortRunHighGrayLevelEmphasis, LowGrayLevelZoneEmphasis, SizeZoneNonUniformity, **SmallAreaEmphasis**, Coarseness

RFE, recursive feature elimination; AdaBoost, adaptive boosting; SVC, support vector classifier; XGB, extreme gradient boosting. The features in bold are commonly selected

### Analysis of selected features

**Fig. 4:**
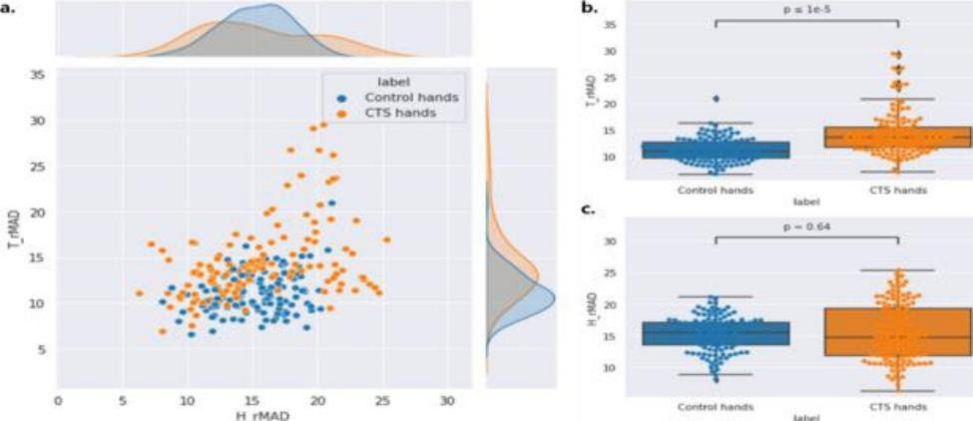
Distribution of robust mean absolute deviation (rMAD) feature values. T_rMAD denotes rMAD obtained from the thenar muscle, and H_rMAD denotes the rMAD obtained from the hypothenar muscle. (**a**) Joint plot of T_rMAD and H_rMAD. (**b**) Distribution of the T_rMAD values within the box plot. (**c**) Distribution of the H_rMAD values with the box plot

**Fig. 5:**
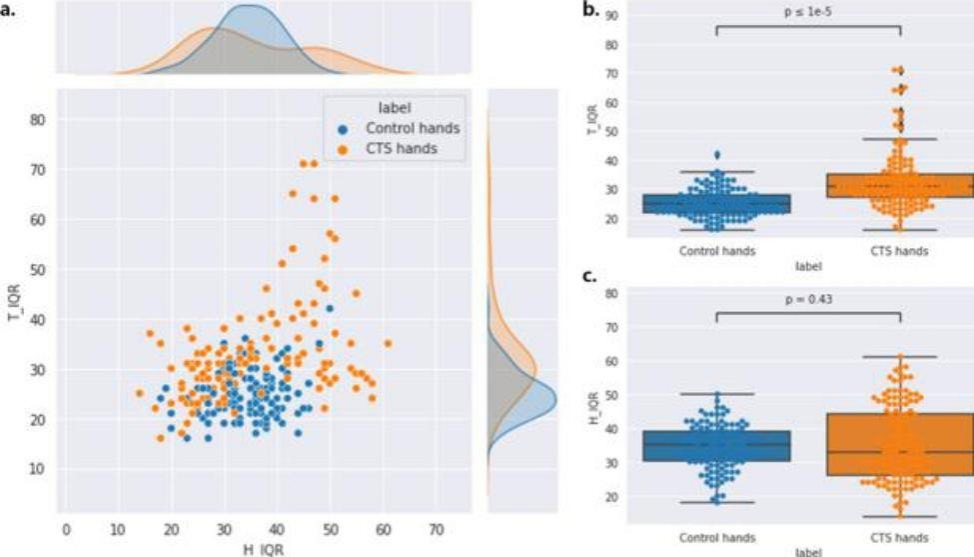
Distribution of interquartile range (IQR) feature values. T_IQR denotes the IQR obtained from the thenar muscle and H_IQR denotes the IQR obtained from the hypothenar muscle. (a) Joint plot of T_IQR and H_IQR. (b) Distribution of the T_IQR values with the box plot. (c) Distribution of the H_IQR values with the box plot

[Supplementary Table 2] presents the definition of rMAD, IQR and small-area emphasis. The rMAD is the mean distance from the gray-level intensity mean, calculated from the intensity values in the range between the 10th and 90th percentiles. IQR represents the range of the 75th and 25th percentile of the image array. The common characteristic of rMAD and IQR is that they are related to robust statistics, because those features exclude outliers by definition. [Figure 4] and [Fig. 5] show the results of rMAD and IQR features analysis which is used to distinguish CTS. As shown in [Fig. 4- and 5-(a)], the distribution of CTS groups for each features had a larger deviation than that of the control group. [(b) and (c) of Figs. 4 and 5], respectively, show that the median values of rMAD and IQR features of the control and CTS groups were similar in the hypothenar region. However, in the thenar region, the median value in the CTS group was significantly higher than that in the control group. Moreover, in the thenar region, there were almost no outliers in the control group, whereas relatively many outliers existed in the hands with CTS.

Lastly, small area emphasis is based on the gray level size zone matrix (GLSZM) features. GLSZM is a statistical representation of a bivariate conditional probability density function of the image distribution values of the gray-level zone, which is the number of adjacent pixels with the same gray-level intensity in the input image. GLSZM is useful for describing heterogeneous nonperiodic texture images and is well suited for the analysis of cell textures with speckle-like structures [11]. Small-area emphasis is a measure of the distribution of small-sized areas, where a large value indicates that there are many small areas with fine textures in the input image. The implication of this features in our study will be explained in the discussion.

## Discussion

In this study, it was shown that the AI assisted quantitative muscle ultrasound analysis improved the accuracy of distinguishing between the hands with CTS and without compared to conventional quantitative analysis. Although conventional quantitative analysis also showed higher EI ratio in the affected hand compared to the unaffected hand, but the AUC, sensitivity and specificity were not high enough. On the other hand, the AUC were significantly improved when ML was applied.

Among the methods for evaluating muscle EI, quantitative analysis is the most sensitive method, and the detection rate for pediatric skeletal muscle disease is > 90% [12, 13], with a sensitivity of approximately 75%, even for diseases that cause less structural muscle abnormality [1]. However, in previous studies that applied this method for CTS diagnosis, Ozsoy-Unubol et al. reported a sensitivity of 71.4% and specificity of 59.4% when using the EI ratio [3]. In another study by Kim et al., the EI ratio had a relatively low AUC (0.66), indicating that its discriminative power was lower than that of muscle disease [2]. This is because the change in muscle EI is more pronounced in myogenic disease than in neurogenic disease, and the study by Sogawa et al. also showed a higher AUC value in the myogenic group than in the neurogenic group when compared with the value obtained in normal subjects [4].

Recently, several studies have been conducted on the use of AI to enhance the diagnostic performance. First, Sogawa et al. performed texture analysis on muscle ultrasound images from 67 patients: 25 in the neurogenic group, 21 in the myogenic group, and 21 in the healthy group. They performed binary classification between each group using five ML-based classifiers (linear discriminant analysis, quadric discriminant analysis, k-nearest neighbors, support vector machine, and random forest) and reported > 70% classification accuracy between each group, and a classification accuracy > 90% between the myogenic and neurogenic groups; however, the neurogenic group in this study included patients with generalized neuropathy, not focal neuropathy such as CTS. Among studies applying ML to CTS diagnosis, Sayin et al. used ML algorithms for 109 patients with CTS and 42 healthy individuals to detect CTS, achieving 91% CTS detection accuracy [14]. This study used electrophysiological findings as a variable but has the disadvantage of causing discomfort to the patient, owing to the invasiveness of the procedure. Park et al. applied ML classifiers to 1037 hands with CTS for categorization according to severity, i.e., mild, moderate, and severe grades [15]. Since demographic factors and ultrasound parameter, such as cross-sectional area and palmar bowing, were analyzed as variables, it required a considerable amount of time to collect the information for analysis. In contrast, in our study, several features could be extracted and analyzed from muscle ultrasound images that could be obtained within 1 min. Therefore, our method is much simpler and is not significantly affected by the operator’s skill.

In our study, we used four ML classifiers: random forest classifier, AdaBoost, linear SVC, and XGB. The classifiers achieved AUC scores of 0.83, 0.80, 0.86, and 0.81, respectively, which are at least 0.04 and up to 0.10 higher than the scores obtained with conventional methods using quantitative analysis. Furthermore, by using RFE, our study not only identified important features for CTS diagnosis among the radiological features of the thenar and hypothenar images but also increased the AUC scores of the classifiers by reducing overfitting. Among the 176 input features, 15 pairs were used each in the thenar and hypothenar muscles, and the AUC score increased by up to 0.89 in the case of linear SVC.

The features selected through RFE were rMAD, IQR, and small-area emphasis. rMAD and IQR suppress outliers and are related to robust statistics. Because these features are commonly selected as important discriminative features, the impact of outliers on classification performance was deemed significant. Indeed, a significant part of the outliers in the muscle ultrasound image is the hyperechoic fibroadipose septa corresponding to the perimysium. Because the perimysium is not a muscle fiber, there is no change in signal intensity depending on whether denervation is present. Therefore, if this region is included in the analysis, the increase in muscle signal intensity due to neurogenic disease may be diluted, and this is why rMAD or IQR, which excludes outliers, is helpful in improving discrimination performance.

The thenar region of the hands with CTS appeared to have more grayish substances with varying levels of intensity and more speckle-like structures in fine patterns than those of the control hands. The implication was that images of the thenar regions of the hands with CTS tend to have a greater small-area emphasis. In the normal muscle, all muscle fibers, except the perimysium, are homogeneous hypoechoic. Because partial denervation occurs as the neurogenic disease progresses, the normal hypoechoic region and denervated hyperechoic region are mixed and the size of the region with similar signal intensity tends to decrease. As an analogy, this is similar to the difference between gravel and sand grains. Accordingly, the small-area emphasis was deemed to be relatively higher in the hands with CTS.

Our study had some limitations. First, muscle ultrasound texture images vary with age or sex, but a subgroup analysis was not performed because of the insufficient number of subjects. Second, because of the insufficiency of data for a finer classification based on the four classes of severity grades, i.e., normal, mild, moderate, and severe, the subjects of this study were limited to the binary classification for control hands and hands with CTS. To overcome these limitations, in a follow-up study, we plan to construct a dataset with a larger number of patients and an even distribution in terms of demographic factors and severity. Finally, since our method used texture feature of the thenar and hypothenar regions, ROIs in the images were manually annotated by clinicians. In our future study, we plan to use a deep neural network (DNN) which automatically extracts features, and does not need the ROI annotation. The DNN-based model is expected to achieve a higher accuracy as well.

In conclusion, this is the first study to use AI-assisted quantitative analysis of muscle ultrasonographic findings in CTS. We propose an ML-based classification using muscle texture features on ultrasound images. We applied RFE to our models to improve CTS classification accuracy and confirmed that the commonly selected features were clinically significant. Among the ML models, linear SVC had the best performance; if RFE was applied, it showed an AUC of 0.89, which is an improvement of 0.13 compared with the conventional quantitative analysis. Therefore, the proposed method could be utilized by physicians as a useful tool to assist in CTS diagnosis and understand the echo patterns observed in the ultrasonography of patients with CTS.

## Electronic supplementary material

Below is the link to the electronic supplementary material.

Supplementary Material 1

Supplementary Material 2

## Data Availability

The datasets generated and/or analysed during the current study are not publicly available due to its proprietary nature and privacy/ethical concerns, but are available from the corresponding author on reasonable request.

## Abbreviations

CTS: Carpal tunnel syndrome
AI: Artificial intelligence
AUC: Area under the ROC curve
EI: Echo intensity
SD: Standard deviation
SVC: Support vector classifier
