# Supplementary material for: Feasibility of artificial intelligence assisted quantitative muscle ultrasound in carpal tunnel syndrome
Source: BMC Musculoskelet Disord. 2023 Jun 27;24:524. doi: 10.1186/s12891-023-06623-3 (PMC10294449; doi:10.1186/s12891-023-06623-3)
Supplement: Supplementary file 1 — Supplementary Material 1 [file 12891_2023_6623_MOESM1_ESM.docx]

| Supplementary table 2. Parameters commonly selected by the RFE method and their definitions. | |
| --- | --- |
| Parameter | Description |
| Interquartile Range | $P_{75}-P_{25}$ |
| Robust Mean Absolute Deviation | $\frac{1}{N_{10-90}}\sum_{i=1}^{N_{10-90}} \left\vert X_{10-90}\left( i \right)-\bar{X}_{10-90} \right\vert$ |
| Small Area Emphasis | $\frac{\sum_{i=1}^{N_{g}} \sum_{j=1}^{N_{s}} \frac{P_{zone}(i, j)}{j^{2}}}{N_{z}}$ |
| RFE, recursive feature elimination. $X$ denotes the input image. $P_{25}$ and $P_{75}$ are the ${25}^{th}$ and ${75}^{th}$ percentiles of the image array, respectively. $N_{10}$ and $N_{90}$ are the numbers of pixels in the ${10}^{th}$ and ${90}^{th}$ percentiles, respectively. $N_{s}$ is the number of discrete intensity values in the image. $N_{z}$ is the number of zones in the input image. | |
