# Supplementary material for: Feasibility of artificial intelligence assisted quantitative muscle ultrasound in carpal tunnel syndrome
Source: BMC Musculoskelet Disord. 2023 Jun 27;24:524. doi: 10.1186/s12891-023-06623-3 (PMC10294449; doi:10.1186/s12891-023-06623-3)
Supplement: Supplementary file 2 — Supplementary Material 2 [file 12891_2023_6623_MOESM2_ESM.docx]

| Supplementary table 1. Baseline characteristics | | |  |  |
| --- | --- | --- | --- | --- |
|  | Hands with CTS (N=47) | Hands without CTS (N=27) | | *p*-value |
| Age (±SD) | 56.15 (±9.13) | 40.30(±13.86) | | *p*<0.001^*^ |
| Male/Female | 17/30 | 9/18 | | *P*=0.806^†^ |
| Hand dominance (Rt/Lt) | 43/4 | 24/3 | | *P*=0.701^‡^ |
| Lesion side (Rt/Lt) | 24/23 |  | |  |
| CTS, carpal tunnel syndrome; SD, standard deviation; *, Mann-Whitney U test; †, Pearson’s chi-squared test; ‡, Fisher's exact test | | | |  |
